# Clinical efficacy and safety of 6-thioguanine in the treatment of childhood acute lymphoblastic leukemia: A protocol for systematic review and meta-analysis

**DOI:** 10.1097/MD.0000000000020082

**Published:** 2020-05-01

**Authors:** Liang Chen, Huai-Xiu Yan, Xiao-Wei Liu, Wen-Xin Chen

**Affiliations:** Department of Hematology, Inner Mongolia Baogang Hospital (The Third Affiliated Hospital of Inner Mongolia Medical University), No. 20 Shaoxian Road, Kundulun District, Baotou, Inner Mongolia Autonomous Region, 014010, China.

**Keywords:** 6-mercaptopurine, 6-thioguanine, childhood acute lymphoblastic leukemia, clinical efficacy, meta-analysis, randomized controlled trials, safety

## Abstract

**Background:**

To systematic review the efficacy and safety of 6-thioguanine (6-TG) in the substitute of 6-mercaptopurine (6-MP) in the treatment for patients with childhood acute lymphoblastic leukemia (ALL) in the maintenance phase, and to explore its clinical application value. It provides theoretical guidance for the maintenance treatment of ALL in children from the perspective of evidence-based medicine.

**Methods:**

By means of computer retrieval, Chinese databases were searched: Chinese Biomedical Database (CBM), China national knowledge internet (CNKI), Chongqing Weipu Database (VIP), and Wanfang Database; Foreign databases: PubMed, The Cochrane Library, Embase, and Web of Science were applied to find out randomized controlled trial (RCT) for 6-TG in childhood acute lymphoblastic leukemia. By manual retrieval, documents without electronic edition and related conference papers were retrieved. The retrieval time ranges from the beginning of the establishment of the databases to September 1st, 2019. According to the inclusion, and exclusion criteria by 3 researchers, the literature screening, data extraction, and research methodological quality evaluation were completed. RevMan 5.3 software was applied to evaluate the quality of the included literature, and Stata 12.0 software was used to conduct meta-analysis of the outcome indicators of the included literature.

**Results:**

This study systematically evaluated the efficacy and safety of 6-TG in the substitute of 6-MP as a maintenance drug for childhood acute lymphoblastic leukemia. Through the key outcome indicators, this study is expected to draw a scientific, practical conclusion for 6-TG in the treatment of childhood acute lymphoblastic leukemia. This conclusion will provide evidence-based medical direction for clinical treatment.

**Conclusion:**

The efficacy and safety of 6-TG in the substitute of 6-MP in the maintenance treatment of childhood acute lymphoblastic leukemia will be confirmed through this study. The conclusions will be published in relevant academic journals.

**Registration:**

PROSPERO (registration number is CRD42020150466).

## 1 Introduction

Leukemia is a group of malignant clonal diseases, which is a malignant tumor of hematopoietic system caused by mutation of the hematopoietic stem cells or hematopoietic progenitor cells. As leukemia cells self-renewal enhances, proliferation is out of control, differentiation is disordered, apoptosis is blocked, and stagnating at different stages of cell development. Abnormal primitive and immature cells (leukemia cells) in bone marrow can proliferate and inhibit normal hematopoiesis. They can infiltrate various organs such as liver, spleen, and lymph nodes. Their clinical manifestations are anemia, bleeding, infection, infiltration, etc.^[1–3]^ At present, the etiology is not completely clear, which may be related to viral infection, physical, and chemical factors, genetic quality, and so on. Its pathogenesis includes the transformation of the proto-oncogene, the aberration of anti-oncogene, inhibition of apoptosis, etc.^[4,5]^ ALL can be divided into acute lymphoblastic leukemia (ALL) and acute myelogenous leukemia (AML) based on the primary cell series involved. Among them, ALL is the most commonly seen malignant disease in childhood, accounting for 30% of children's tumors.^[6,7]^ More than 50 years ago, the survival rate of leukemia was meagre, and leukemia was considered to be a refractory disease. However, the cure rate of ALL in children (defined as disease-free survival for more than 10 years) can reach as high as 80%, which is related to stratified therapy based on the risk of relapse, and biological characteristics of leukemia cells, increasingly excellent supportive treatment and communication, and the optimization of treatment regimens by cooperative research at home and abroad.^[8,9]^ Among them, according to morphology, immunology, cytogenetics, molecular biology, early treatment response, and other risk factors, the risk classification of ALL children with corresponding stratified chemotherapy provided have got more importance, and attention from clinicians increasingly.^[10]^

ALL in children is the most common malignant hematological neoplasm. The key to its treatment is to kill tumor cells and keep the children in remission effectively. Although the cure rate of ALL in children is over 90%, the prognosis of ALL in children is not ideal, and they even face a recurrence risk of up to 20%.^[11]^ Mercaptopurine drugs as part of the continuous treatment of ALL have been the key to prevent its recurrence for a long time. Among them, 6-mercaptopurine (6-MP) is used in the period of maintenance therapy, while 6-thioguanine (6-TG) is only used in the period of intensive treatment. Generally speaking, there is no optimal drug regimen in the treatment process.^[12,13]^ For more than 60 years, 6-MP has been used to treat ALL in children. Its mechanism has not been fully elucidated. The treatment-related hepatotoxicity and bone marrow suppression are still significant challenges for clinicians. Due to the lack of direct parameters to inspect the efficacy of patients, it is difficult to grasp the intensity of treatment. Clinicians still need to further adjust the treatment regimen to reduce the resistance to mercaptopurine drugs.^[14]^ Since 1980, pharmacokinetic studies have found that 6-TG has a more direct intracellular activation pathway, shorter cytotoxicity time, and stronger effect than 6-MP, which clarify why 6-TG is more potentially useful theoretically. Since 1990, clinical studies have been conducted to compare the efficacy of the 2 drugs. Some experimental animal data also preliminarily show that 6-TG may be more effective than 6-Mp.^[15]^ Therefore, we use a systematic review method to study the efficacy, and safety of 6-TG as an alternative to 6-MP in the maintenance phase of childhood acute lymphoblastic leukemia for the clinical treatment of childhood acute lymphoblastic leukemia, which provides a scientific basis for clinical treatment of childhood acute lymphoblastic leukemia.

## 2 Methods

### 2.1 Study registration

PROSPERO (registration number is CRD42020150466). The registered website for this protocol is https://www.crd.york.ac.uk/PROSPERO/.

### 2.2 Document inclusion and exclusion criteria

#### 2.2.1 Types of research

Randomized controlled trial of 6-TG in the treatment of childhood acute lymphoblastic leukemia. Whether or not the blind method and distribution concealment are mentioned, there is no restriction on the language of literature.

#### 2.2.2 Research object

Inclusion criteria:

(1) Literature published at home and abroad on 6-TG in the substitute of 6-MP for treating ALL in children.
(2) The subjects of study were children with acute lymphoblastic leukemia (from 1 to 18 years old), excluding patients with acute biphenotypic leukemia, severe complications, or complications with no limitation of race and nationality.
(3) Intervening measures were suffered children who were given 6-TG or 6-MP at the end of induction remission.

Exclusion criteria:

(1) Non-RCT research literature.
(2) Duplicated published and reported literature.
(3) Only abstracts, missing raw data, or the valid unattained data even contacting the author.
(4) Documents that interfere with the drug in this study before the diagnosis of acute lymphoblastic leukemia in children.
(5) Animal experiments.
(6) Review literature.
(7) Repeated literature.

#### 2.2.3 Intervening measures

The experimental group (6-TG group) was treated with chemotherapeutic drugs and supportive therapy based on 6-TG. The control group (6-MP group) was treated with chemotherapeutic drugs and supportive therapy based on 6-MP. The included drug dosage and mode of use of each study may vary.

#### 2.2.4 Outcome indicator

Main outcome indicators

(1) Overall efficiency;
(2) The recurrence rate of the isolated central nervous system (ICNS);
(3) The recurrence rate of non-ICNS (the recurrence rate of bone marrow, testis, and other parts, the multi-site recurrence exclusion);
(4) Overall recurrence rate;
(5) Incidence of secondary malignant tumors;
(6) Incidence of adverse events;

Secondary outcome indicators

(1) Incidence of hepatic venous occlusion;
(2) Ki-67;
(3) Fucosyltransferase-4 (FUT4);
(4) Suppressor of Cytokine Signaling-3 (SOCS-3);
(5) Serum matrix metalloproteinase (SMMP), interleukin (IL), tissue-specific inhibitor-2 (TIMP-2), and prostaglandin E2 (PGE-2);
(6) Hospitalization days;
(7) The levels of leukocyte (WBC), platelet (PLT), and hemoglobin (Hb);
(8) Liver function (alanine aminotransferase, aspartate aminotransferase, γ glutamyl transferase);
(9) Lymphocyte subpopulation (CD3, CD4, CD8, CD4/CD8, CD19);
(10) Coagulation function.

#### 2.2.5 Data extraction

Literature screening: Firstly, we led the retrieved titles into NoteExpress 2.0 software, which is document management software, and set up a catalog database. Secondly, the software was used to classify and sort out the initial documents, and duplicated documents were removed by using the function of automatic searching for duplicated documents by software. Thirdly, 2 researchers screened the literature independently. By reading the topics and abstracts of each study, the literature that did not meet the inclusion criteria was excluded. Fourthly, the full texts of the corresponding the literature of the remaining titles were downloaded. The full text was read with the combination of the inclusion, and the criteria were excluded from determining whether the selected literature can be included. Documents with incomplete information can be obtained by contacting the original author through e-mail. All excluded documents should record the reasons for the exclusion. Fifthly, the results of literature screening were cross-checked by 2 researchers. If there are differences, they will consult with the third party; if it cannot be solved after discussion, it will be dealt with in consultation with the third party.

### 2.3 Search strategy

Computer Search Chinese Database: CMB, China national knowledge internet (CNKI), Chongqing Weipu Database (VIP), and Wanfang Database; Foreign Database: PubMed, The Cochrane Library, EMbase, and Web of Science. The randomized controlled trial (RCT) study of 6-TG in the treatment of children's ALL and the included references, academic conferences and network resources, etc in the literature were inquired at the same time to find out the research that may meet the inclusion criteria. The retrieval time is from the establishment of databases to September 1, 2019.

Firstly, the clinical problems were refined by the principle patient, intervention, contrast, outcome, study (PICOS)^[16]^:

P: children with acute lymphoblastic leukemia;
I: chemotherapy;
C: 6-thioguanine vs 6-mercaptopurine;
O: effectiveness and safety;
S: RCTs.

Chinese search terms include (Chinese pinyin): “Ertong”, “Xiaohai”, “Ji-xing-lin-ba-xi-bao-xing-bai-xue-bing”, “6-liuniaopiaoling”, “2-anjipiaoling-6(1H)-liutong”, “6-qiupiaoling”, “6-qiujipiaoling”, “Lejining”, and “Sui-ji-dui-zhao-shi-yan”. English search terms include: “child”, “children”, “precursor cell lymphoblastic leukemia–lymphoma”, “acute lymphoid leukemia”, “6-thioguanine”, “thioguanin”, “6-mercaptopurine”, “mercaptopurine”, “randomized trial”, “RCT”. Finally, the method of combining keywords and free words is used for further supplementary retrieval. The retrieval strategy takes the PubMed database as an example, as shown in Table 1.

**Table 1:**
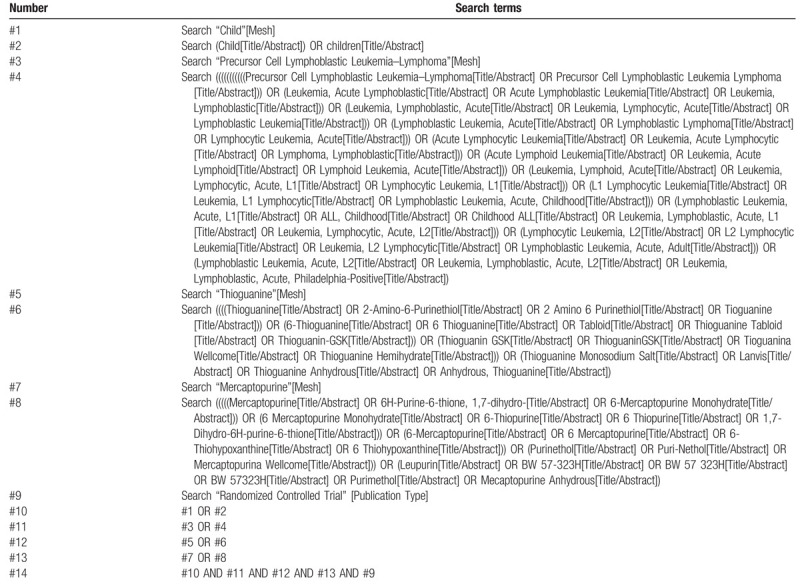
Search strategy for the PubMed database.

### 2.4 Data extraction

Firstly, data extraction tables are designed according to research purposes and requirements. The contents of data extraction tables include:

(1) Basic information: the title, publication time, publishing magazines, publishing languages, authors, research sites, etc of the included literature.
(2) The characteristics of the study included: demographic characteristics, pathological types, stages, intervention measures of treatment group and control group, etc.
(3) Inclusion of information related to literature bias risk assessment.
(4) Extraction of relevant data of outcome indicators.

**Figure 1:**
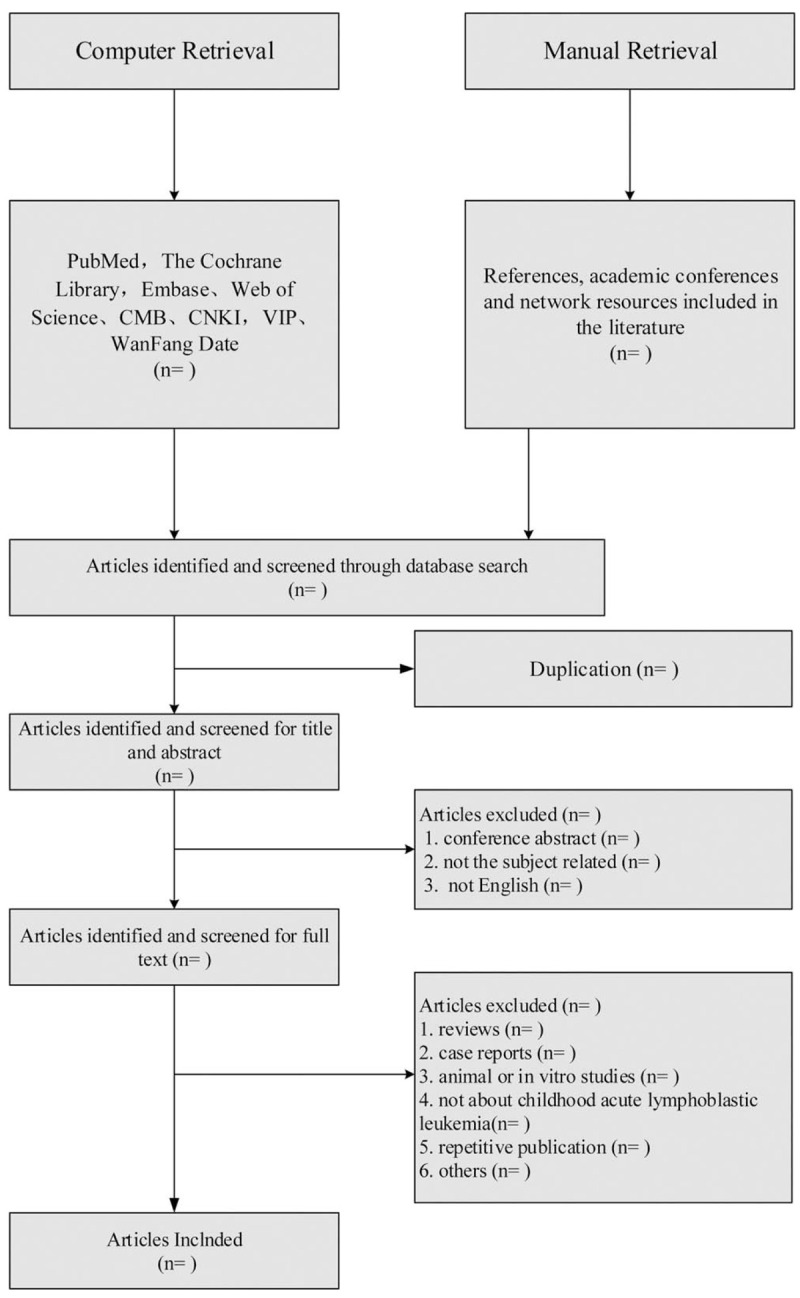
Flowchart of literature selection.

Secondly, to ensure the accuracy of data extraction, 2 researchers independently extract relevant data based on the data extraction table with the cross-check to make statistical analysis after making it accurate and unambiguous. In case of contradictory opinions, they can be settled through consultation with the third party. When coming across the data missing, the author of the relevant literature was contacted through e-mail to obtain the relevant original data. For the duplicate publications of the same study, the most comprehensive one was selected for data entry. The literature screening process is shown in Figure 1.

### 2.5 Quality assessment

According to the “Bias Risk Assessment” tool recommended by Cochrane Collaboration Network (Version 5.1.0), the 6 aspects of selective bias (random sequence generation and allocation concealment), implementation bias (blind method for subjects and experimenters), measurement bias (blind method for outcome assessors), follow-up bias (incomplete outcome data), reporting bias (selective reporting of results), and other bias were used respectively to make the evaluation for the quality of evidence for inclusion in research.^[17]^ According to the criteria in Cochrane Handbook 5.1.0, “High Risk”, “Low Risk”, “Unclear” were used to express the evaluation results. When evaluating the quality of evidence, the 2 researchers independently review the evaluation results. If there is any difference of opinion, it should be settled through consultation with the third party. RevMan 5.3 software was used to draw the bias risk pictures.

### 2.6 Statistical method

#### 2.6.1 Statistical analysis

The software of Review Manager 5.3 and STATA12.0 provided by Cochrane was applied to make statistical analysis for the included literature, and forest maps, and funnel maps were made from the results. Relative risk (RR) and standardized mean difference (SMD) were applied as the effect combination indicator. Various effect quantities were expressed by 95% confidence interval (CI), and there was the significant difference between the results of *P* < .05.

#### 2.6.2 Heterogeneity test

Before the meta-analysis of the literature that meets the inclusion requirements, the heterogeneity of the literature was tested by statistics at first. In recent years, Ka Fang test has been widely used with the application of an *I*^2^ index to make the heterogeneity testing of included literature research.^[18]^ The size of heterogeneity among the studies is measured by the percentage of *I*^2^. If the value of *I*^2^ is less than 25%, it shows that the heterogeneity among the studies is small, while if the value of *I*^2^ is between 25% and 50%, it indicates that there is moderate heterogeneity among the studies. Fixed effect model can be used to merge the research data. If the value of *I*^2^ is more than 50%, it is considered that there is a high degree of heterogeneity among the studies. Sensitivity analysis or subgroup analysis is needed to identify the sources of heterogeneity among the literature. In this paper, the combination of research data adopts the way of stochastic effect model.

### 2.7 Sensitivity analysis

Sensitivity analysis refers to the important factors that influence the results of the study, such as, inclusion criteria, randomized grouping, loss or withdrawal of the study subjects, different statistical methods, criteria for evaluating efficacy and selection of efficacy (e.g., ratio or relative risk), etc to observe the homogeneity between the studies, or whether the final results of the synthesis can be changed so as to determine whether the results of the study are stable. If the results of sensitivity analysis are the same or similar to those of this meta-analysis, it shows that the results of the study are reliable. If the results of sensitivity analysis are quite different from those of meta-analysis in this paper, it shows that the results of meta-analysis have potential factors that influence the effectiveness of interventions. Therefore, it is necessary to draw cautious conclusions or only make a descriptive analysis.

### 2.8 Subgroup analysis

This study will apply the subgroup analysis method to find out the causes of heterogeneity. Subgroup analysis will be applied from the following aspects, including, dosage, dosage form, frequency of drug use, duration of drug use, ethnic differences, and so on.

### 2.9 Publication bias

Bias refers to the difference between inferred results, and true values, which can be drawn from various stages of clinical trials, including selective bias, implementation bias, measurement bias, follow-up bias, and reporting bias. The first 4 biases can be reduced by controlling the quality of the included literature; however, reporting bias is mainly assessed by assessing publication bias. According to the number of studies included, when the number of studies exceeds 10 items, the funnel plot method will be used to evaluate the bias qualitatively.^[19]^ When the number of studies is less than 10 items, the quantitative evaluation is carried out by using the Egger linear regression method with STATA 12.0 software. It defines that when *P* < .05, there is publication bias.

### 2.10 Ethics and dissemination

The ethical approval of clinical research is not suitable for this study.

## 3 Discussion

ALL is a malignant disease originating from abnormal proliferation of B and T cells in the bone marrow. The lymphocyte proliferation of ALL is uncontrolled, and cannot differentiate, mature, and function. Tumor cells infiltrate into bone marrow and other hematopoietic tissues and organs, and inhibit the normal hematopoietic function of bone marrow, resulting in related clinical symptoms. ALL can occur in all age groups, mainly seen in children, and adolescents. It ranks first in the incidence of malignant tumors in children. ALL belongs to a highly heterogeneous disease. Different clonal subtypes have different biological characteristics, and the clinical efficacy, and prognosis are different as well.^[20]^

In recent years, with the development of immunology, cytogenetics, molecular biology, and other disciplines, more in-depth understanding of the pathogenesis and clinical characteristics of ALL has been achieved, and its treatment strategies have made new progress as well. In addition to the main treatment of chemotherapy, hematopoietic stem cell transplantation and targeted therapy have been applied as well.^[21]^ However, for children with ALL, the status of chemotherapy is not questionable. The prognosis of children with ALL has been greatly improved thanks to the treatment strategy guided by risk classification, and optimized combination of chemotherapeutic drugs.

Clinical use of thioguanine drugs in children with ALL is complicated in dose, course of treatment, drug selection, and remedy of toxic and side effects. The therapeutic effect of ALL was also linked to the disease grade, gender, and age of the patients. All the included studies were based on the risk classification of children with intrathecal injection of chemotherapeutic drugs in the prophylactic treatment of isolated central nervous system leukemia. Currently, there is no evidence proving that 6-TG is better than 6-MP in terms of improving the quality of life, prolonging the survival time, and reducing the mortality of ALL children. This study provides some important clues for the drug selection of thioguanine drugs during maintenance therapy of ALL in children.^[22]^ There is no definite clinical effect of 6-TG in the substitute of 6-MP in the treatment of ALL in children during maintenance therapy. Further studies are needed to provide evidence-based evidence for the clinical treatment of 6-TG. Therefore, it is suggested that the monitoring of active metabolite concentration and side effects in blood should be strengthened in the treatment of 6-TG. Anti-infection, liver protection, jaundice alleviation, and low molecular weight heparin can be offered to improve the prognosis of children.^[23]^ In terms of preventing the recurrence of ALL in children, 6-TG may be more effective than 6-MP, but 6-TG has strong hepatotoxicity, which should be avoided to use for in the long run. Ongoing pharmacogenetic studies can establish models to explore whether children with specific type can benefit from 6-TG.

The efficacy and safety of 6-TG in the substitute of 6-MP in the maintenance phase of childhood acute lymphoblastic leukemia (CALL) are controversial due to the lack of high-quality clinical studies. Therefore, there are some controversies about the effectiveness and safety of 6-TG at present. As a result, this study evaluates the efficacy and safety of 6-TG in the substitute of 6-MP as a maintenance drug to treat childhood acute lymphoblastic leukemia through a comprehensive search of related studies. This study will draw a scientific and practical conclusion through a systematic review.

## Author contributions

**Conceptualization:** Liang Chen, Wen-Xin Chen.

**Data curation:** Huai-Xiu Yan, Xiao-Wei Liu.

**Formal analysis:** Liang Chen, Huai-Xiu Yan.

**Funding acquisition:** Wen-Xin Chen.

**Methodology:** Liang Chen, Huai-Xiu Yan.

**Software:** Huai-Xiu Yan, Xiao-Wei Liu.

**Writing – original draft:** Liang Chen, Huai-Xiu Yan, Xiao-Wei Liu.

**Writing – review & editing:** Wen-Xin Chen.
